# The “hype” of hydrops in classifying vestibular disorders: a narrative review

**DOI:** 10.1007/s00415-020-10278-8

**Published:** 2020-11-17

**Authors:** Marly F. J. A. van der Lubbe, Akshayaa Vaidyanathan, Vincent Van Rompaey, Alida A. Postma, Tjasse D. Bruintjes, Dorien M. Kimenai, Philippe Lambin, Marc van Hoof, Raymond van de Berg

**Affiliations:** 1 Department of Otolaryngology and Head and Neck Surgery, Maastricht University Medical Center, Maastricht, The Netherlands; 2 The D-Lab, department of Precision Medicine, GROW research institute for Oncology, Maastricht University, Maastricht, The Netherlands; 3 Department of Otorhinolaryngology and Head and Neck Surgery, Antwerp University Hospital, Faculty of Medicine and Health Sciences, University of Antwerp, Antwerp, Belgium; 4 Department of Radiology and Nuclear Medicine, Maastricht University Medical Center, Maastricht, The Netherlands; 5 Department of Otorhinolaryngology, Gelre Hospital, Apeldoorn, The Netherlands; 6 Central Diagnostic Laboratory, Maastricht University Medical Center, Maastricht, The Netherlands; 7 Research and Development, Oncoradiomics SA, Liege, Belgium; 8 School for Mental Health and Sciences, Maastricht University, Maastricht, The Netherlands; 9 Department of Otorhinolaryngology, Leiden University Medical Center, Leiden, The Netherlands

**Keywords:** Vestibular disorders, Menière’s, Disease, Symptoms, Endolymphatic hydrops, Distinctiveness, Magnetic resonance imaging

## Abstract

**Background:**

Classifying and diagnosing peripheral vestibular disorders based on their symptoms is challenging due to possible symptom overlap or atypical clinical presentation. To improve the diagnostic trajectory, gadolinium-based contrast-enhanced magnetic resonance imaging of the inner ear is nowadays frequently used for the in vivo confirmation of endolymphatic hydrops in humans. However, hydrops is visualized in both healthy subjects and patients with vestibular disorders, which might make the clinical value of hydrops detection on MRI questionable.

**Objective:**

To investigate the diagnostic value of clinical and radiological features, including the in vivo visualization of endolymphatic hydrops, for the classification and diagnosis of vestibular disorders.

**Methods:**

A literature search was performed in February and March 2019 to estimate the prevalence of various features in healthy subjects and in common vestibular disorders to make a graphical comparison between healthy and abnormal.

**Results:**

Of the features studied, hydrops was found to be a highly prevalent feature in Menière’s disease (99.4%). Though, hydrops has also a relatively high prevalence in patients with vestibular schwannoma (48.2%) and in healthy temporal bones (12.5%) as well. In patients diagnosed with (definite or probable) Menière’s disease, hydrops is less frequently diagnosed by magnetic resonance imaging compared to the histological confirmation (82.3% versus 99.4%). The mean prevalence of radiologically diagnosed hydrops was 31% in healthy subjects, 28.1% in patients with vestibular migraine, and 25.9% in patients with vestibular schwannoma. An interesting finding was an absolute difference in hydrops prevalence between the two diagnostic techniques (histology and radiology) of 25.2% in patients with Menière’s disease and 29% in patients with vestibular schwannoma.

**Conclusions:**

Although the visualization of hydrops has a high diagnostic value in patients with definite Menière’s disease, it is important to appreciate the relatively high prevalence of hydrops in healthy populations and other vestibular disorders. Endolymphatic hydrops is not a pathognomic phenomenon, and detecting hydrops should not directly indicate a diagnosis of Menière’s disease. Both symptom-driven and hydrops-based classification systems have disadvantages. Therefore, it might be worth to explore features “beyond” hydrops. New analysis techniques, such as Radiomics, might play an essential role in (re)classifying vestibular disorders in the future.

**Electronic supplementary material:**

The online version of this article (10.1007/s00415-020-10278-8) contains supplementary material, which is available to authorized users.

## Introduction

The diagnosis of peripheral vestibular disorders highly relies on a symptom-driven classification system [1]. Peripheral vestibular disorders are a group of heterogeneous conditions with a possible complex symptom presentation and substantial overlap in clinical features [2]. Although a variety of audiological and vestibular tests complement the clinical diagnosis, objective biomarkers are generally missing. This leads to diagnostic challenges in many peripheral vestibular disorders, for instance, in Menière’s disease (MD).

MD is a condition of the inner ear characterized by spontaneous episodes of vertigo, fluctuating sensorineural hearing loss, aural fullness, and tinnitus. The disease is strongly associated with the post-mortem, histological finding known as endolymphatic hydrops (EH). EH is considered to be the underlying pathology of MD [3]. The histologic confirmation of EH is not possible in a clinical setting. Therefore, the diagnosis of MD mainly depends on symptom-based criteria [4]. Symptom-based criteria, however, poorly capture preclinical disease states or atypical clinical presentation [5]. Recently, the role of imaging in vestibular disorders changed, as the application of gadolinium-based contrast-enhanced magnetic resonance (MR) imaging of the inner ear enabled the in vivo visualization of EH [6]. Nowadays, some clinics use the combination of symptomatology and *hydrops imaging* to improve their diagnostic trajectory for vestibular disorders [7], with emphasis on MD. The development of *hydrops imaging* also resulted in the proposal of new classification methods for vestibular entities like MD. Motivated by the goal to enable a disease description more closely related to the underlying pathology, researchers proposed the concept of *hydropic ear disease* [8]. Importantly though, the role of EH in the pathophysiology of vestibular disorders is still not fully understood. Next to this, EH is also found in subjects without any symptoms of MD [9]. It has been proposed that EH is merely an epiphenomenon or byproduct of another (unknown) process [3, 9]

At this point, the added value of classifying vestibular patients and distinguishing them from healthy individuals based on the presence or absence of EH is not fully clear. Therefore, to evaluate the value of *hydrops imaging*, in combination with symptomatology, for vestibular classification and diagnostics, this narrative review aimed to graphically map and cluster symptoms and the occurrence of EH, based on their relative prevalence in healthy individuals and patients with common peripheral vestibular disorders. A secondary aim was to explore the diagnostic value of EH in addition to symptom classification in patients suspected for MD.

## Methods

### Search strategy and study selection

Multiple literature search strategies in *PubMed* and *Google Scholar* were performed in February and March 2019 to identify papers reporting the prevalence of EH and/or neuro-otologic symptoms in three types of study populations:

1. “Diseased” populations: including patients diagnosed with a vestibular disorder as recognized by the 10th revision of the International Statistical Classification of Diseases and Related Health Problems (ICD10).
2. “General” populations: non-preselected populations reflecting both patients with vestibular disorders and healthy subjects.
3. “Healthy reference” populations: including subjects without a vestibular history.

The search strategy consisted of electronic database searching, reference checking, and citation searching. A variety of MeSH (Medical Subject Headings) terms and free-text keywords were combined to conduct multiple search strategies in *PubMed* (see “Supplementary materials”). Based on title and abstract, one reviewer (ML) selected and summarized English published studies deemed relevant within the scope of this review. To complement the initial search, the reference lists of the primary selected studies, as well as the “cited by” feature of Google Scholar [10], were used to screen for additional studies to include. This process was iterated until no new eligible studies were identified. A comprehensive literature overview was composed without any selection restrictions.

### Data collection

The following data items were tabulated from the selected publications: *Author*, *Year of publication, study design, recruitment strategy and definition of the study population, sample size, age, EH assessment techniques, symptom description,* and *study outcomes.* To explore the diagnostic value of EH in patients with MD, additional data were collected from the studies that investigated the diagnostic role of EH in patients with MD. The following items were collected: sensitivity, specificity, positive predictive values, and negative predictive values.

### Estimation of EH and symptom prevalences

Differences in study characteristics were identified and processed after the consultation of a second reviewer (MH). The minimal criteria for the combined assessment of the collected data were: similarities in recruitment strategy and definition of the study populations, study outcomes, and EH assessment technique. To homogenize study outcomes, point prevalences, frequency rates, and period prevalences up to 1-year were combined. These were regarded to indicate best the proportion of a population that has active symptoms.

Although several EH assessment techniques exist [11, 12], this review combined the results of the most commonly reported grading method for hydrops MR evaluation in literature [13, 14]. This method categorizes hydrops in either “mild” or “significant.” Hydrops prevalences reported with the lowest cut-off point (mild) for cochlear and/or vestibular hydrops [13] were combined for prevalence estimation.

Results of studies with outlying age distributions or small sample sizes (*n* < 7) were disregarded for prevalence estimation. The mean prevalence of symptoms and EH was estimated in patients with vestibular disorders, the general population, and the healthy reference population.

### Quality assessment

To determine the reliability of the estimated prevalences and to allow better transparency of the results in this review, a quality assessment of data synthesis was performed based on five predefined criteria (for the reliability criteria see “Supplementary materials”). For each criterion that was met, a score of one point was allocated. The reliability of the mean prevalences was estimated to be high (5) or low (1).

### Graphical clustering

To graphically cluster symptom and EH occurrence based on their relative prevalence in vestibular patients and health, the estimated prevalences were plotted in a four-dimensional (x, y, r, α) bubble chart using the open-source, interactive graphing library for Python, Plotly 3.10.0.[15].

The estimated prevalence of symptoms and EH in patients diagnosed with a vestibular disorder and healthy reference populations were plotted on the x-axis and y-axis, respectively. The estimated prevalence in the general population was displayed by the radius (r) of the bubbles: large bubbles reflected common symptoms. The reliability of the results was presented by the transparency of the bubbles in which transparent bubbles represented a low level of reliability, and opaque bubbles, a high level of reliability.

## Results

### Study selection

In total, 69 publications were selected after database searching and citation and reference retrieval for estimating the prevalence of EH and neuro-otologic symptoms in different types of study populations [3, 9, 12, 18–34, 36–84]. Based on the available data, three vestibular disorders were selected for prevalence estimation: (1) The clinical diagnosis of Menière’s disease based on diagnostic criteria (Table 1) [16], (2) The diagnosis of vestibular schwannoma confirmed by MRI, (Table 2), and (3) The clinical diagnosis of vestibular migraine based on diagnostic criteria (Table 3) [17]. Other vestibular diagnoses, such as benign paroxysmal positional vertigo and acute unilateral vestibulopathy, were not reviewed since no EH imaging studies in relation to these vestibular disorders were found. No post-mortem studies were found published on the prevalence of EH in patients with vestibular migraine. Due to the lack of contrast-enhanced imaging studies in patients with a vestibular schwannoma, two studies were selected that used non-contrast-enhanced MRI to diagnose EH [18, 19].

**Table 1:** The estimated prevalences in unilateral and bilateral Menière's disease

Feature	Description^a^	Sample sizes^b^ (n)	Age^c^ (year)	Estimated prevalence (%)	Range^d^	Reliability^e^	References
Headache	Headache N.O.S.	55–119	> 19	64.1	41.2–81	3	[40–42]
Tinnitus	Tinnitus N.O.S.	55–501,306	> 37	82.1	59–91.4	2	[40, 41, 43–45]
Dizziness**	Dizziness N.O.S.	–	–	100	–	1	-
Hearing loss	Any subjective difficulty with hearing	55–1376	> 37	76.4	55–93	2	[40, 41, 43, 45]
Otalgia	Otalgia N.O.S.	55	–	17	–	1	[41]
Aural fullness	Aural fullness N.O.S.	37–726	> 20	73.4	65–80.6	3	[40, 41, 44–46]
Migraine	Migrainous headaches	37–119	> 18	27.4	8.4–51	2	[40, 41, 45, 47]
Vertigo	Vertigo N.O.S.	37	> 20	100	–	1	[44]
Falls	Falls without external factors	501,306	> 37	14.6	13.7–15.4	2	[43]
EH (histology)	Endolymphatic hydrops in any part of the labyrinth except for the apical turn	28–165	–	99.4	98.8–100	3	[3, 9]
EH (radiology)	Mild cochlear or vestibular hydrops	18–396	> 7	82.3	44.4–100	3	[12, 25–31, 33, 34, 39, 48–51]
EH (radiology)	Mild cochlear or vestibular hydrops in the asymptomatic contralateral ear	23–198	> 7	27.6	8.6–65	3	[25–34]
EH* (radiology)	Mild cochlear or vestibular hydrops in “possible” MD	7–122	> 16	77.7	41.1–100	3	[31, 36–38]

Data were not available

*N.O.S.* Not otherwise specified

^a^Symptom and hydrops description. In case of heterogenous or missing descriptions, the feature was defined as N.O.S

^b^Lower and upper limits of the combined sample sizes

^**c**^Lower limit of the combined age samples

^d^Range of the combined prevalences

^e^The reliability of the combined results defined by a 5-point scoring system: 5 = high reliability, 1 = low reliability

^*^Hydrops prevalences in patients with only one symptom like hearing loss or isolated vertigo spells (cochlear or vestibular MD)

^**^Dizziness was estimated to be 100% prevalent among patients with Menière’s disease since vertigo was 100% prevalent

**Table 2:** The estimated prevalences in patients diagnosed with vestibular schwannoma

Feature	Definition^a^	Sample sizes^b^ (n)	Age^c^ (year)	Estimated prevalence (%)	Range^d^	Reliability^e^	References
Headache	Headache N.O.S.	122–541	> 13	25.2	18–36	4	[52–56]
Tinnitus	Tinnitus N.O.S.	122–541	> 13	63.8	56–83	3	[52–56]
Dizziness	Dizziness N.O.S.	126–206	> 17	33.3	23–48	2	[53, 55, 56]
Hearing loss	Any subjective or objective hearing loss	122–541	> 13	91	85–97	3	[52–56]
Otalgia	Otalgia N.O.S.	126	> 17	12	–	1	[56]
Aural fullness	Ear pressure N.O.S.	541	Adults	28	–	2	[54]
Migraine	Migraine (diagnostic criteria)	122	> 13	4.9	–	1	[52]
Vertigo	Vertigo N.O.S.	122–206	> 13	24.4	5,3–49	2	[52, 53, 55]
CPA tumor (radiology)	Requirement for diagnosis	–	–	100	–	1	–
EH (histology)	Distension of Reissner’s membrane in vestibule or cochlea	11–12	> 44	48.2	36.4–60	2	[21, 24]
EH* (radiology)	Cochlear or vestibular hydrops	13–183	> 26	25.9	21–30.8	1	[18, 19]

Data were not available

*N.O.S.* Not otherwise specified

^a^Symptom and hydrops definition. In case of heterogenous or missing descriptions, the feature was defined as N.O.S

^b^Lower and upper limits of the combined sample sizes

^c^Lower limit of the combined age samples

^d^Range of the combined prevalences

^e^ The reliability of the combined results defined by a 5-point scoring system: 5 = high reliability, 1 = low reliability

^*^EH was detected with non-contrast-enhanced MR imaging

**Table 3:** The estimated prevalences in patients diagnosed with vestibular migraine

Feature	Description^a^	Sample sizes^b^ (n)	Age^c^ (year)	Estimated prevalence (%)	Range^d^	Reliability^e^	References
Headache	Headache N.O.S.	71–84	-	97.1	95.2–99	2	[40, 41]
Tinnitus	Tinnitus N.O.S.	16–252	> 19	44.8	26–55	1	[40, 41, 57, 58]
Hearing loss	Any subjective difficulty with hearing	71–252	> 19	32.5	14–89	2	[41, 57, 58]
Otalgia	Otalgia N.O.S.	71	–	27	–	1	[41]
Aural fullness	Aural fullness N.O.S.	16–252	> 19	37.6	12–70	2	[40, 41, 57, 58]
Migraine	Migrainous headaches	16–84	Adults	80	56–100	2	[40, 41, 58]
Vertigo	Sensation of spinning, swaying or tilting	252	> 19	73	–	1	[57]
EH (radiology)	Mild cochlear or vestibular hydrops	7–60	> 26	28.1	0–85.7	1	[12, 36, 59]

Data were not available

*N.O.S.* Not otherwise specified

^a^Symptom and hydrops definition. In case of heterogenous or missing descriptions, the feature was defined as N.O.S

^b^Lower and upper limits of the combined sample sizes

^c^Lower limit of the combined age samples

^d^Range of the combined prevalences

^e^The reliability of the combined results defined by a 5-point scoring system: 5 = high reliability, 1 = low reliability

### Study characteristics and data collection

The characteristics of the individual studies are presented in the supplementary materials.

Within the selected publications, symptom descriptions varied, and were sometimes absent or not specified. The definition of EH also varied between different post-mortem studies. Four post-mortem studies defined EH as a displacement of Reissner’s membrane or the membranous walls in the vestibule [3, 9, 20, 21]. Other studies only examined EH in the cochlear turns and did not explicitly define EH in the vestibule [22–24]. All contrast-enhanced imaging studies used the same semi-qualitative assessment technique [13] and the lowest cut-off point (mild) for grading EH.

### Estimation of EH and symptom prevalence

The results of the estimation of symptom and EH prevalences, together with the quality assessment of data synthesis for study populations of the three investigated vestibular disorders, are shown in Tables 1, 2, 3. Overall, neuro-otologic symptoms were more prevalent in vestibular disorders compared to healthy references with no vestibular history, as demonstrated in Tables 1, 2, 3, 4, 5.

**Table 4:** The estimated prevalences in the general population

Feature	Description^a^	Sample sizes^b^ (*n*)	Age^c^ (years)	Estimated prevalence (%)	Range^d^	Reliability^e^	References
Headache	Headache N.O.S.	> 200	> 3	41.1	16–60.2	4	[60–62]
Tinnitus	Tinnitus N.O.S.	4083–15,445	> 14	18.6	17–20.3	4	[63, 64]
Dizziness	Dizziness N.O.S.	1003–3267	> 18	18.5	16.7–24.2	5	[64–67]
Hearing loss	Any objective hearing loss > 25 dB	3267–7490	> 18	12.9	7.2–16.1	5	[66, 68–70]
Otalgia	Otalgia not associated with infection or otitis	411	> 25	12.5	–	1	[71]
Aural fullness	Aural fullness not associated with infection	411–866	> 25	12.4	7–17.8	2	[72, 73]
Migraine	Migraine (diagnostic criteria)	> 200	> 3	10.5	10–11	4	[60–62]
Vertigo	Rotational, positional or recurrent vertigo	1003–4869	> 18	8.4	4.9–15.2	4	[64, 65, 74]
Falls	Falls without external factors	2394–3267	> 40	5.5	1.5–11.5	5	[64, 66, 67]
CPA tumor (radiology)	Cerebellopontine angle tumor on MRI	2000	> 45	0.2	–	1	[75]
EH (histology)	Distension of Reissner’s membrane in vestibule or cochlea	560–703	> 0	14.3	9–19.5	4	[20, 76]

Data were not available

*N.O.S.* Not otherwise specified

^a^Symptom and hydrops descriptions. In case of heterogeneous or missing descriptions, the feature was defined as N.O.S

^**b**^Lower and upper limits of the combined sample sizes

^**c**^Lower limit of the combined age samples

^d^Range of the combined prevalences

^e^The reliability of the combined results defined by a 5-point scoring system: 5 = high reliability, 1 = low reliability

**Table 5:** The estimated prevalences in the healthy references with no vestibular history

Feature	Description^a^	Sample sizes^b^ (*n*)	Age^c^ (year)	Estimated prevalence (%)	Range^d^	Reliability^e^	References
Headache*	Headache N.O.S.	–	–	20.1	–	1	[77]
Tinnitus	Tinnitus N.O.S.	78–501,306	> 10	20.4	8.1–32.5	2	[43, 78, 79]
Hearing loss	Any subjective difficulty with hearing	78–501,306	> 37	26.2	25.4–27	3	[43, 79]
Dizziness	Dizziness N.O.S.	78–282	> 10	17	16–18	3	[79, 80]
Otalgia	Otalgia not associated with infection or otitis	1387	> 18	25.5	–	1	[81]
Aural fullness	Aural fullness not associated with infection	78	> 10	12	–	1	[79]
Migraine	Migraine (diagnostic criteria)	501,306	> 37	2.9	–	2	[43]
Vertigo	Rotational, positional or recurrent vertigo	326–368	> 11	4.9	3.5–7.3	3	[78, 80]
Falls	Falls without external factors	282–501,306	> 37	3.8	1–6.6	4	[43, 80]
CPA tumor (radiology)	Cerebellopontine angle tumor on MRI	24,246–46,414	> 20	0.05	0.02–0.07	4	[82, 83]
EH (histology)	Distension of Reissner’s membrane in the cochlea	24–118	> 0	12.5	3.5–17	3	[20, 22, 23]
EH (radiology)	Mild cochlear or vestibular hydrops	22–32	> 20	31	0–100	3	[25, 27, 29, 39, 84]

Data were not available

*N.O.S.* Not otherwise specified

^a^Symptom and hydrops definition. In case of heterogenous or missing descriptions, the feature was defined as N.O.S

^b^Lower and upper limits of the combined sample sizes

^**c**^Lower limit of the combined age samples

^d^Range of the combined prevalences

^e^The reliability of the combined results defined by a 5-point scoring system: 5 = high reliability, 1 = low reliability

*Headache prevalence in the healthy population was estimated by subtracting the percentage of headaches associated with dizziness from the overall prevalence of headache in the general population

The mean prevalence of histologically diagnosed EH ranged from 12.5% in healthy references to 99.4% in patients with MD. Radiologically diagnosed EH prevalences ranged from 25.9% in patients with vestibular schwannoma to 82.3% in patients with MD. The EH prevalence ranges varied from narrow (98.8–100%) to extreme broad (0–100%) demonstrated in Tables 1 and 5 by the prevalence ranges of EH histologically diagnosed in MD patients and radiologically diagnosed in healthy references, respectively. The reliability of the prevalence estimations varied from low (1) to high (5) (median = 2, IQR = 2). The prevalence estimations in the general population were most reliable (median = 4, IQR = 1.5) compared to the prevalence estimations in patients with vestibular migraine (median = 1.5, IQR = 1).

### Clustering of features

**Fig. 1:**
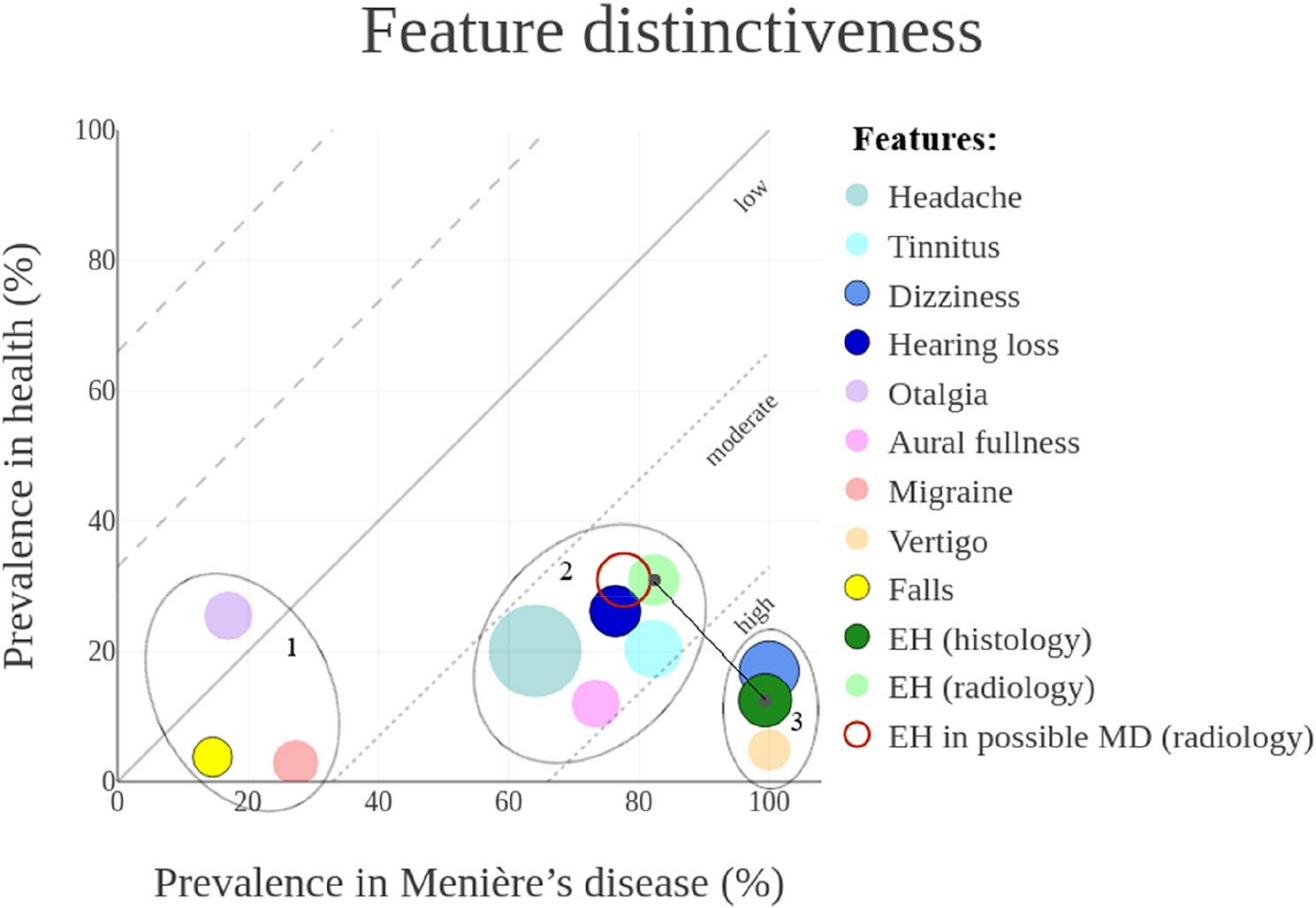
Graphical representation of feature distinctiveness in a four-dimensional (x,y,r,α) bubble chart. Estimated prevalences in patients diagnosed with MD and in healthy references were plotted on the x-axis and y-axis, respectively. This resulted in a low, moderate, or high level of distinctiveness. The overall feature prevalence in both health and disease were presented as bubble size (r). The reliability of the results were presented by transparency (α). Features plotted under the reference line were distinctive for MD. The absolute distance between EH (histology) and EH (radiology) represented the discrepancy in distinctiveness between the two techniques

**Fig. 2:**
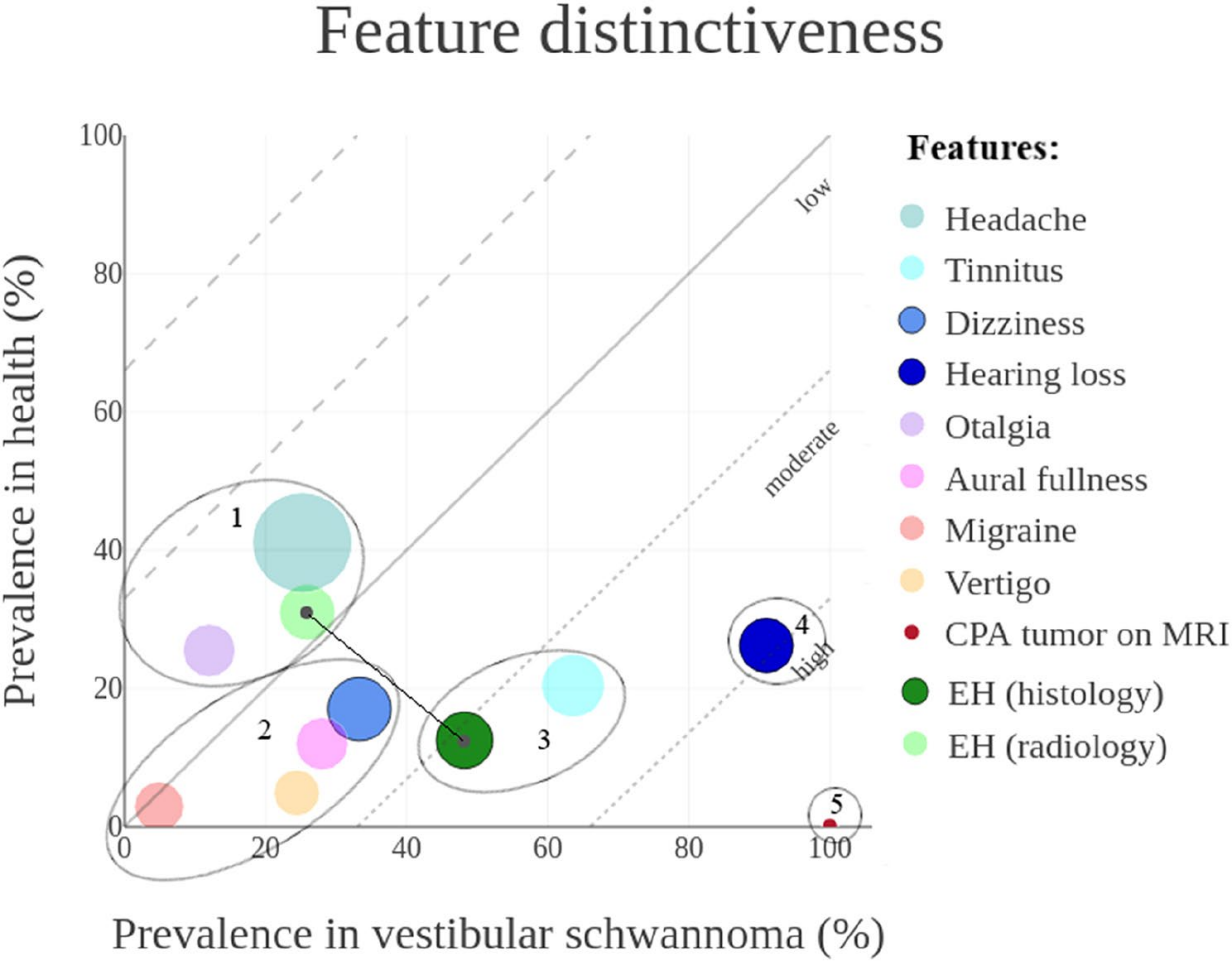
Graphical representation of feature distinctiveness in a four-dimensional (x,y,r,α) bubble chart. Estimated prevalences in patients diagnosed with vestibular schwannoma and healthy references were plotted on the x-axis and y-axis, respectively. This resulted in a low, moderate, or high level of distinctiveness. The overall feature prevalence in both health and disease were presented as bubble size (r). The reliability of the results was presented by transparency (α). Features plotted under the reference line were distinctive for vestibular schwannoma. The absolute distance between EH (histology) and EH (radiology) represented the discrepancy in distinctiveness between the two techniques

**Fig. 3:**
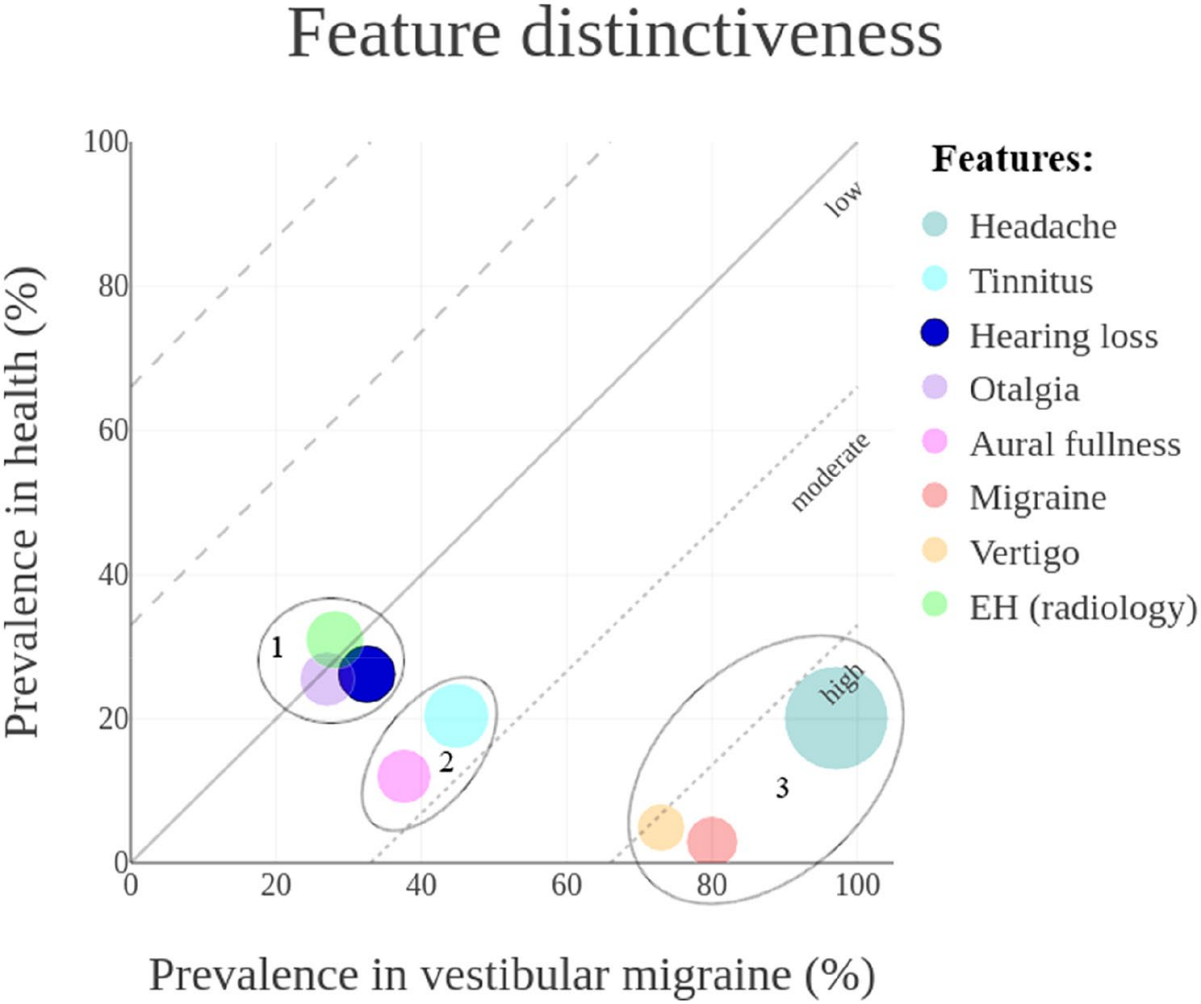
Graphical representation of feature distinctiveness in a four-dimensional (x, y, r, α) bubble chart. Estimated prevalences in patients diagnosed with vestibular migraine and healthy references were plotted on the x-axis and y-axis, respectively. This resulted in a low, moderate, or high level of distinctiveness. The overall feature prevalence in both health and disease were presented as bubble size (r). The reliability of the results were presented by transparency (α). Features plotted under the reference line were distinctive for vestibular migraine

Figures 1, 2, 3 show the mean prevalence of symptoms and EH, in three vestibular diagnoses plotted against the prevalence in healthy references. Consequently, different clusters of features arose at different locations in the figures. Clusters with similar prevalences on the x-axis and y-axis were encircled. Clusters of features plotted above the oblique reference line are more prevalent in healthy references, and clusters plotted below the oblique line are more prevalent in the vestibular disorder. The greater the distance between the plotted features and the reference line, the more the feature distinguishes between a physiological condition and pathological condition.

#### *In Menière’s disease*

Three different clusters were identified based on ascending prevalences, as shown in Fig. 1. The features in the first cluster distinguished the least, and the features in the third cluster distinguished the best between disease and health, accordingly. In patients with MD, *migrainous headaches* and *Falls* occurred more often in comparison with healthy references (27.4% vs. 2.9% and 14.6% vs. 3.8%, respectively). The otologic symptoms, together with *headaches,* and the radiological diagnosis of EH, were common in patients with MD (ranged from 64.1 to 82.3%) compared to healthy individuals (ranged from 12 to 31%). Subjective *hearing loss* was not always present in patients with MD (76.4%) despite that audiometrically documented hearing loss is required for the diagnosis of MD. The most specific and best distinctive features were *vertigo* and histologically confirmed EH (100% vs. 4.9% and 99.4% vs. 12.5%, respectively).

A separate analysis of the prevalence of radiologically diagnosed EH on the contralateral side of patients with unilateral MD resulted in a mean prevalence of 27.6% (ranged from 8.6 to 65%) [25–34]. Contrast-enhanced imaging studies also assessed the prevalence of EH in patients who presented with only one symptom like hearing loss or isolated vertigo spells. This group was addressed as ‘possible MD’ [16, 35] and the mean prevalence of EH (77.7%) [31, 36–38] was similar to the prevalence in patients diagnosed with ‘definite or probable MD’ (82.3%).

#### *In vestibular schwannoma*

Five different clusters were identified based on the estimated prevalences in healthy references and patients with a vestibular schwannoma, as shown in Fig. 2. Although five times more prevalent in patients than in healthy references, only a quarter of the patients with vestibular schwannoma experienced *vertigo* (24.4% vs. 4.9%). In regard to other symptoms, *hearing loss* and *tinnitus* were more common in vestibular schwannoma compared to the healthy reference population (91% vs. 26.2% and 63.8% vs. 20.4%, respectively). In comparison with healthy references, EH was often histologically confirmed in patients with vestibular schwannoma (48.2% vs. 12.5%). The prevalence of EH in patients with vestibular schwannoma was lower when diagnosed with MRI compared to the post-mortem diagnosis (25.9% vs. 48.2%, respectively). As expected, a specific and highly distinctive feature in patients with vestibular schwannoma was the finding of a cerebellopontine angle tumor on MRI (100% in vestibular schwannoma patients vs. 0.05% in healthy references).

#### *In vestibular migraine*

Three different clusters were identified based on the ascending prevalences, as shown in Fig. 3. *Otalgia*, *hearing loss* and the radiological diagnosis of EH in patients with vestibular migraine clustered based on similar prevalence in patients and healthy references (27% vs. 25.5%, 32.5% vs. 26.2% and 28.1% vs. 31%, respectively). *Tinnitus* and *aural fullness* were more common in patients (44.8% vs. 20.4% and 37.6% vs. 12%, respectively). *Headaches*, including *migrainous headaches*, and *vertigo* were most specific and distinctive in the diagnosis of vestibular migraine with extensive differences in prevalence between patients and healthy references (97.1% vs. 20.1%, 80% vs. 2.9%, and 73% vs. 4.9%, respectively).

#### Comparison between the vestibular disorders

Although unique clusters were identified for every vestibular diagnosis, an overlap of symptom clusters, based on their prevalences, was found. The symptoms that were most prevalent in MD also occurred in approximately 20% of the patients with vestibular schwannoma (*vertigo and aural fullness*) and 40% of patients with vestibular migraine (*tinnitus, hearing loss and aural fullness*). *Vertigo* occurred in around 73% of VM patients.

The estimated prevalence of EH differed between the two diagnostic techniques (histology and radiology). In healthy references, the radiological diagnosis of EH occurred more often compared to the histological diagnosis (31% vs. 12.5%). In patients with MD and vestibular schwannoma, EH was less frequently diagnosed by imaging compared to the post-mortem confirmation (82.3% vs. 99.4% and 25.9% vs. 48.2%, respectively). The absolute difference in EH prevalence between the two diagnostic techniques was 25.2% in patients with MD and 29% patients with vestibular schwannoma as calculated by the following formula:

$${ \sqrt {\left( {x_{2} - x_{1}} \right)^{2} + \left( {y_{2} - y_{1}} \right)^{2} }}$$

### The diagnostic performance of hydrops imaging in Menière’s disease

An overview of the results of three publications that investigated the diagnostic role of EH observed on contrast-enhanced MRI in patients with MD is shown in Table 6. All studies used the same method for grading EH [13] and had similar study protocols. (For further study details see “Supplementary materials”). Next to this, the diagnostic performances of three other image features related to MD, “vestibular endolymphatic space contacting the oval window,” the degree of “perilymphatic enhancement,” and “Saccule to utricle ratio,” were presented.

**Table 6:** An overview of test performance of hydrops imaging in the diagnosis of Menière’s disease

Semi-quantitative grading method [13]				Features beyond hydrops	
Author, year, reference				Author, year, reference	
Conte, 2019 [25]	MD (*n* = 27) vs. Healthy (*n* = 24)			Conte, 2019 [25]	MD (*n* = 27) vs. Healthy (*n* = 24)
Vestibular EH	SE: 74 (53–88)	Cochlear EH	SE: 74 (53–88)	Marker:	SE: 81 (61–93)
“Mild”	SP: 83 (62–95)	“Mild”	SP: 96 (77–100)	“VESCO”	SP: 96 (77–100)
	PPV: 83(62–95)		PPV: 95 (74–100)		PPV: 96 (76–100)
	NPV: 74 (53–88)		NPV: 77 (57–89)		NPV: 82 (62–93)
Yoshida, 2018 [27]	MD (*n* = 52) vs. Healthy (*n* = 42)			Bernaerts, 2019 [85]	MD (*n* = 78) vs. Contralateral (*n* = 78)
Vestibular EH	SE: 94	Cochlear EH	SE: 87	Marker:	SE: 85
“Mild”	SP: 93	“Mild”	SP: 62	addition of “Cochlear PE”	SP: 92
	PPV: 94		PPV: 74		
	NPV: 93		NPV:79		
Vestibular EH	SE: 77	Cochlear EH	SE: 71	Attyé, 2017 [39]	MD (*n* = 30) vs. Healthy (*n* = 30)
“Significant”	SP:100	“Significant”	SP: 91	Marker	SE: 50
	PPV: 100		PPV: 90	“SURI”	SP:100
	NPV: 78		NPV: 72		
Attyé, 2017 [39]	MD (*n* = 30) vs. Healthy (*n* = 30)				
Vestibular EH	SE: 77	Cochlear EH	SE: 60		
“Mild”	SP: 10	“Mild”	SP: 33		
Vestibular EH	SE: 47	Cochlear EH	SE: 37		
“Significant”	SP: 70	“Significant”	SP: 87		

*SE* sensitivity, *SP* specificity, *PPV* positive predictive value, *NPV* negative predictive value, *VESCO* vestibular endolymphatic space contacting the oval window, *PE* perilymphatic enhancement, SURI Saccule to utricle ratio inversion

The sensitivity of the semi-qualitative grading method [13] for “mild” vestibular or cochlear hydrops was reported from 60 to 94%. The specificity was reported from 10 to 96%. The positive predictive value was reported from 74 to 95% and the negative predictive value from 74 to 93%. When shifting the cut-off value to the grading “significant” hydrops, the sensitivity and the negative predictive value decreased, while the specificity and positive predictive value increased [27, 39]. The sensitivities of the other image features ranged from 50 to 85% and their specificities from 92 to 100%.

## Discussion

This narrative review aimed to graphically map neuro-otologic symptoms and EH based on their prevalence in common peripheral vestibular disorders and health, to analyze their value for vestibular classification and diagnostics. To the best of the authors’ knowledge, this narrative review was first in providing an estimation of feature distinctiveness based on prevalences. Point and period prevalences were combined to estimate the mean prevalence.

The results showed that amongst various features, EH is more prevalent in MD than in other vestibular disorders. Therefore, *hydrops imaging*, in combination with symptomatology, is expected to be a promising diagnostic tool in MD. Though, EH also has a relatively high prevalence in healthy temporal bones (12.5%) and patients with vestibular schwannoma (48.2%) as well. Therefore, EH is not specific for MD, and detecting EH, although valuable, should not directly indicate the diagnosis of MD. Next to this, the discrepancy found in the prevalences of histological and radiological diagnoses of EH, might suggest an inaccuracy in detecting EH with MR imaging. This implies that MR evaluation of EH faces challenges in recognizing (non)-pathological or (not-) clinically relevant variants.

### Symptomatology in vestibular disorders

In all three vestibular disorders studied, depending on prevalence, different and distinctive clusters of symptoms emerged. As expected in patients with vestibular schwannoma *hearing loss*, and *tinnitus* were the most common symptoms; while in patients with vestibular migraine *headaches,* and *vertigo* were more common. In patients with MD, as expected, *vertigo*, *hearing loss*, *tinnitus,* and *aural fullness* were most common. Nonetheless, *headaches* were also quite frequent.

These symptom clusters can be used to differentiate between vestibular disorders. However, the results of this review demonstrated four difficulties regarding symptom-based classifications. First, neuro-otologic symptoms occur, in small prevalences, in healthy reference populations. Although symptoms are often considered to be a derivative of pathological states, they can occur outside the direct context of a (vestibular) disease. Second, within a defined pathological state, symptom prevalences can range vastly. These ranges may be caused by the various symptom definitions used in the reviewed publications. Next to this, patients often find it hard to describe their (dizzy) symptoms [86], which might also explain the extensive ranges in symptom prevalences. Third, only 75% of the patients with MD experience subjective hearing loss despite the obligatory audiometrically documented hearing loss for the diagnoses of MD. Perhaps vertigo complaints dominate the perception of hearing loss. Fourth, the expression of auditory symptoms (*hearing loss*, *tinnitus,* and *aural fullness*) in patients with vestibular migraine and the presence of *headaches* in a subset of patients with MD suggest there is a substantial symptom overlap between the two entities. It is uncertain whether this overlap is due to the inclusion of incorrectly diagnosed subjects in the study populations, or that the clinical phenotypes resemble each other [40]. This raises the question whether other classifications are possible or even more appropriate. Symptoms are subjective, prone to terminology problems [1], and they potentially overlap within vestibular entities [40] and are, therefore, not always optimal for classifying vestibular disorders. The use of additional (bio) markers would be helpful.

### Hydrops as histological marker in vestibular disorders

The presence of EH as a histological marker for inner ear disorders has been proposed [9, 87]. Especially in MD, EH is considered to have important diagnostic value since it has been histologically demonstrated in almost every case of MD [3, 9].

The results of this review reaffirmed the potential diagnostic value of EH as a histologic marker in discriminating patients with MD from healthy persons. However, it is important to highlight the fact that EH is also found in normal temporal bones. Apical hydrops occurs in 15% of the human cochleae but is assumed to be of no pathologic significance [88]. The prevalence of EH in other parts of the healthy labyrinth is poorly documented but seems to occur sporadically [20].

Furthermore, EH is found in almost 50% of the patients with vestibular schwannoma and is also well documented in other neuro-otologic conditions such as otosclerosis, chronic otitis media, labyrinthitis, and Mondini dysplasia [87, 89]. Therefore, EH is not a pathognomic feature and the probability of finding hydrops in an otologic population seems considerable. EH may be a valuable marker for MD, but cannot provide a definitive diagnosis without the support of clinical data [14]. Confirming EH should, therefore, not directly indicate the diagnosis of MD.

### Histological versus radiological diagnoses of EH

Recent developments in contrast-enhanced MR imaging enabled the radiological diagnosis of EH in vivo. Its diagnostic value has been broadly investigated and debated in the literature [14]. A striking finding of this review was the discrepancy in prevalence existing between the histological and radiological diagnoses of EH, documented in MD and vestibular schwannoma. It might be hypothesized that EH is better visible in a more advanced disease stage when examining post-mortem, given that the severity of EH seems to increase with the duration of MD [90]. However, this does not explain the relatively higher prevalence of radiologically confirmed EH compared to histologically confirmed EH in healthy references (31% vs. 12.5%). It might be possible that the radiological diagnosis of EH contributes to more false-positive results in healthy subjects and more false-negative results in patients with vestibular disorders. This implies that the discrepancy in prevalence could be attributable to a radiological inaccuracy in demonstrating EH, which seems plausible given the numerous influencing factors such as the MR sequences, image resolution, and interobserver variability [6, 14, 91, 92]. Also, the extensive ranges of the radiological diagnosis of EH found in this review justify the assumption of a radiological inaccuracy of approximately 25% with respect to the post-mortem examination. However, it is important to acknowledge that, although histology is seen as gold standard for confirming hydrops, this technique might also be influenced by the presence of artifacts.

Although *hydrops imaging* successfully identifies EH in subjects with definite MD resulting in relatively high sensitivity (60%–94%) [25, 27, 39], “mild” hydrops is consistently reported in other vestibular disorders and even in healthy subjects. Using *hydrops imaging* as a diagnostic test might result in false-positive outcomes leading to the overdiagnosis of MD. As a diagnosis of MD might imply medical treatment or even surgical intervention, having a diagnostic test with high specificity and positive predictive value is critical. However, studies that investigated the diagnostic role of EH in patients with MD reported a wide range in test specificity (10–96%), despite comparable study protocols. The reproducibility of the semi-qualitative grading technique can, thus, be questioned.

As shown in Table 6, increasing the cut-off value towards significant hydrops will decrease the false-positive results and improve specificity, but it will decrease the sensitivity. *Hydrops imaging* seems to not yield optimal diagnostic performance on its own, though in combination with symptomology, it could play an important role in the diagnosis of MD.

### The challenges of hydrops imaging in vestibular disorders

MR imaging is nowadays the most commonly used method to detect EH in clinic [7]. The high prevalence of EH (82.3%) detected in patients with MD underlines the potential diagnostic value of hydrops imaging. Nevertheless, multiple challenges are related to correctly interpreting the value of the presence of EH.

The first challenge is to determine whether EH is clinically relevant. MR imaging detected EH in approximately 28% of the contralateral asymptomatic MD ears. Comparable rates in autopsy series were found [39]. This suggests that EH occurs in symptomatic and asymptomatic forms. If *hydrops imaging* would be able to identify clinically relevant but ‘silent’ ears, it would be extremely valuable for the early detection, treatment, or even prevention of vestibular disorders in the future. However, it is still uncertain whether asymptomatic hydrops is a preclinical disease state or not. The percentages found in MR imaging could correlate with 35% incidence of patients developing bilateral MD within ten years [93]. However, it might as well correlate with the 31% of EH found in healthy subjects [14, 25, 27, 29, 39, 84].

The second challenge is to determine whether the found EH is equal to a pathological state:

The endolymphatic space seems to vary highly between healthy subjects [39, 84]. EH in healthy individuals might be a deviation of a personal baseline but might also be a physiological variation of normal anatomy. One could hypothesize that EH in principal is an asymptomatic process, and/or that a personal threshold needs to be exceeded to become symptomatic. It is also possible that EH is merely a byproduct of inner ear damage [9] and, although more prevalent, is not always indicative for pathology.

The third challenge is regarding implications for clinicians. It is uncertain how to handle the group of patients who present with only one symptom like hearing loss or isolated vertigo spells [31]. After all, the time delay between symptom onset of hearing loss and vertigo can reach up to more than five years [31]. The prevalence of EH in this group did not substantially differ (77.7%) from the prevalence in patients diagnosed with definite or probable MD (82.3%). Yet, it is unclear whether these patients should be diagnosed and treated the same as patients with the full-blown symptom spectrum of MD based on the presence or absence of EH.

The challenges mentioned above arise since the phenomenon of EH is not fully understood. EH may be an entity on its own, causing the clinical syndrome of MD, but also other processes (e.g., EH as a byproduct, EH as an asymptomatic process, EH as an own entity “Hydropic ear disease”) might be applicable [8].

Next to the challenges mentioned above, it should be noted that several disadvantages are related to contrast-enhanced MR imaging. Although contrast agents have not shown evidence of ototoxicity [6, 94], leakage into the cerebral spinal fluid and deposits in brain tissue have been reported [95]. Moreover, contrast-enhanced MR imaging is not feasible for every patient due to contrast allergy, renal failure, and impaired labyrinthine enhancement after IT injection [6]. A non-invasive imaging technique would be preferable.

### Limitations

The methodology of this review was constrained by the approach of the search strategy and the subjective aspect of study selection, quality assessment, generalization, and synthesis of the collected data. This hampers the reproducibility of this review.

The high level of heterogenicity in the collected studies did not allow a traditional meta-analysis. Furthermore, this review has not excluded any publications based on their quality or study limitations. The collected data from the selected studies were liable to suffer from selection bias. These limitations may have influenced the results of the prevalence estimation. Therefore, the mean prevalences in this review should not be interpreted as definitive epidemiologic data. The purpose of this narrative review was not to provide a methodologically and statistically robust model but to generate a first concept on clustering symptoms and EH according to their relative prevalences in health and vestibular disorders.

### Features beyond hydrops: future perspectives

This review focused on the most commonly used grading method for hydrops MR evaluation [13, 14]. With this grading method, *hydrops imaging* seems not to yield optimal diagnostic performance [25, 27, 39]. No consensus has been reached on how to assess the radiological finding of EH. Therefore, other parameters like the “Saccule to utricle ratio,” “vestibular endolymphatic space contacting the oval window,” and the degree of “perilymphatic enhancement” were also investigated and showed promising results regarding test specificity, as shown in Table 6. [25, 39, 85]. This implies that it might be worth to explore features “beyond” hydrops. After all, other processes might also be related to inner ear disorders. Recently, an exploratory study detected differences in image features extracted from conventional MR images between patients with MD compared to controls [96]. The approach of extracting and analyzing quantitative image features, which are sometimes not perceptual for the human eye, is referred to as *Radiomics* [97, 98]. Physicians often rely on the presence of objective abnormalities to diagnose disease, for example, EH. However, in case of the absence of objective pathology, it is not always correct to rule out disease. Unknown, not (yet) perceptible factors may play a crucial role in disease development and have yet to be discovered. Techniques like *Radiomics* and morphological-based assessment [25, 39, 85] of the labyrinth may play an essential role in the future in (re)classifying and diagnosing vestibular disorders.

This review could serve as a foundation for future studies that eventually will allow reclassification of peripheral vestibular diseases. It can also serve as an encouragement to explore new fields of image analysis (e.g., *Radiomics*) that will exceed the reach of the human eye.

## Conclusions

The combination of symptomatology and hydrops imaging is valuable in the diagnosis of MD. However, it is important to appreciate the probability of finding hydrops in both patients with vestibular disorders and healthy individuals. Based on prevalences, this review showed that EH is not a pathognomic feature and that the diagnosis of EH does not directly indicate the diagnosis of MD.

In order to optimize the classification and the diagnostic trajectory of peripheral vestibular disorders, it is essential to acknowledge the challenges and disadvantages that come with symptom-driven classification systems and the “hype” of hydrops imaging. It could be valuable to focus “beyond” hydrops and to explore other approaches such as *Radiomics*, as well.

## Electronic supplementary material

Below is the link to the electronic supplementary material.

Supplementary file1 (XLSX 245 kb)

Supplementary file2 (DOCX 33 kb)
