# Supplementary material for: The “hype” of hydrops in classifying vestibular disorders: a narrative review
Source: J Neurol. 2020 Nov 17;267(Suppl 1):197–211. doi: 10.1007/s00415-020-10278-8 (PMC7718205; doi:10.1007/s00415-020-10278-8)
Supplement: Supplementary file 2 — Supplementary file2 (DOCX 33 kb) [file 415_2020_10278_MOESM2_ESM.docx]

**Supplementary materials**

1. **Supplementary methods**

1a. Search strategy and study selection

For database searching, six groups of keywords were defined. Both MeSH (Medical Subject Headings) terms, as well as free-text terms, were used as keywords.

Group of keywords

1. Neuro-otologic symptoms:

Keywords: *‘Symptoms’, ‘Dizziness’, ‘Vertigo’, ‘Oscillopsia’, ‘drop attacks’, ‘falls’, ‘Hearing loss’, ‘Tinnitus’, ‘Aural fullness’, ‘Ear Pressure’, ‘Otalgia’, ‘Headache’, ‘Migraine’*

1. Patient population diagnosed with a vestibular disease (ICD10)

Keywords: *‘Meniere’s disease’, ‘Benign paroxysmal vertigo’, ‘Vestibular neuronitis’, ‘Labyrinthitis’, ‘Vestibular Schwannoma’, ‘Vestibular migraine, ‘Superior canal dehiscence syndrome’, ‘Benign paroxysmal positional vertigo’, ‘Bilateral vestibulopathy’*

1. Healthy population defined as a population with no vestibular history:

Keywords: *‘Healthy’, ‘Healthy volunteers’, ‘Healthy controls’, ‘Controls’*

1. The general population reflecting both healthy subjects and patients

Keywords: *‘General population’, ‘Community’*

1. Confirmation of endolymphatic hydrops (Histology or Radiology)

Keywords: *‘Endolymphatic hydrops’, ‘Endolymphatic space’, ‘Temporal bone’, ‘Histology’ ‘Pathology’, ‘Autopsy’, ‘Post-mortem examination’, ‘Imaging’, ‘Magnetic Resonance Imaging’*

1. Additional terms:

Keywords: *‘Epidemiology’, ‘Prevalence’, ‘Incidence’, ‘Questionnaire’, ‘Survey’*

Within and across each group, keywords were combined using ‘or’/‘and’ hereby conducting multiple searches strategies in PubMed. Two examples of search strategies were:

1. *(((("Meniere Disease"[tiab]) OR "Meniere Disease"[Mesh])) AND (("Vertigo"[tiab]) OR "Vertigo"[Mesh])) AND ((((("Epidemiology"[tiab]) OR "Epidemiology"[Mesh])) OR (("Prevalence"[tiab]) OR "Prevalence"[Mesh])) OR (((("Surveys and Questionnaires"[Mesh])) OR "Surveys"[tiab]) OR "Questionnaires"[tiab]))*
2. *((((("Endolymphatic space") OR "Endolymphatic Hydrops"[tiab]) OR "Endolymphatic Hydrops"[Mesh])) AND ((((("Healthy controls") OR "Controls") OR "Healthy") OR "Healthy Volunteers"[tiab]) OR "Healthy Volunteers"[Mesh])) AND ((("Magnetic Resonance Imaging"[tiab]) OR "Magnetic Resonance Imaging"[Mesh]) OR "Imaging")*

1b. Reliability criteria

In order to determine the reliability of the estimated prevalences, a quality assessment of data synthesis was performed. For each criterion that was met, a score of one point was allocated. For some symptoms, only one eligible data source was found, and a score of 1 was automatically allocated. The reliability was determined to be high (5) or low (1). To graphically represent the reliability of prevalence estimation, the total score was determined by the sum of the allocated scores in the general, the healthy reference, and one of the vestibular disorders populations (Meniere’s disease, Vestibular migraine, and Vestibular schwannoma). A high level of reliability (total score of > 9) was represented in the figures as an opaque bubble. A low level of reliability (total score of < 9) was represented in the figures as a transparent bubble.

**Reliability criteria:**

1. The estimated prevalence was based on multiple references.
2. The estimated prevalence was based on at least one reference with a sample size of more 500 subjects.
3. The range of the extracted prevalence data from the combined studies was less than 20.
4. Feature description was comparable across the combined publications.
5. Subject recruitment and definition of the study populations were comparable across the combined studies.

**2. Supplementary Tables**

1. Tables of study details of tables 1–5 are found in the supplementary tables 1–5 (Supplementary.xlsx)

2. Table of Study details of table 6.

|  | **Study population** | **Imaging** | **Observers** | **Hydrops Grading Method** |
| --- | --- | --- | --- | --- |
| Conte 2019 [25] | Definite MD according to AAO-HNS (n=27)  Contralateral side of patients with SSHL (n=24) | 3T MRI, 3D FLAIR, IV contrast gadoteridol | A senior neuroradiologist and a radiology resident | Semi-quantitative grading method according to Nakashima et al.  **Marker**: vestibular endolymphatic space contacting the oval window (VESCO) |
| Yoshida, 2018  [27] | Definite MD according to AAO-HNS (n=41)  Non otological patients (n=21) | 3T MRI, 3D FLAIR, IV contrast gadodiamide hydrate | A single (blinded) radiologist | Semi-quantitative grading method according to Nakashima et al. |
| Attyé, 2017  [39] | Definite MD according to AAO-HNS (n=30)  Healthy volunteers (n=30) | 3T MRI, 3D FLAIR,  IV contrast gadoterate megluminate | Two head- and neck radiologists | Semi-quantitative grading method according to Nakashima et al.  **Marker**: the saccule to utricle ratio inversion |
| Bernaerts, 2019 [85] | Definite or probable MD according to the in 2015 revised criteria (n=148) vs. contralateral ears. | 3T MRI, 3D FLAIR, IV contrast | Three (blinded) experienced head and neck radiologists | Semi-quantitative grading method according to Baráth et al.  **Marker**: Cochlear PE |
